# Supplementary figures and images for: Genome-wide analyses supported by RNA-Seq reveal non-canonical splice sites in plant genomes
Source: BMC Genomics. 2018 Dec 29;19:980. doi: 10.1186/s12864-018-5360-z (PMC6310983; doi:10.1186/s12864-018-5360-z)

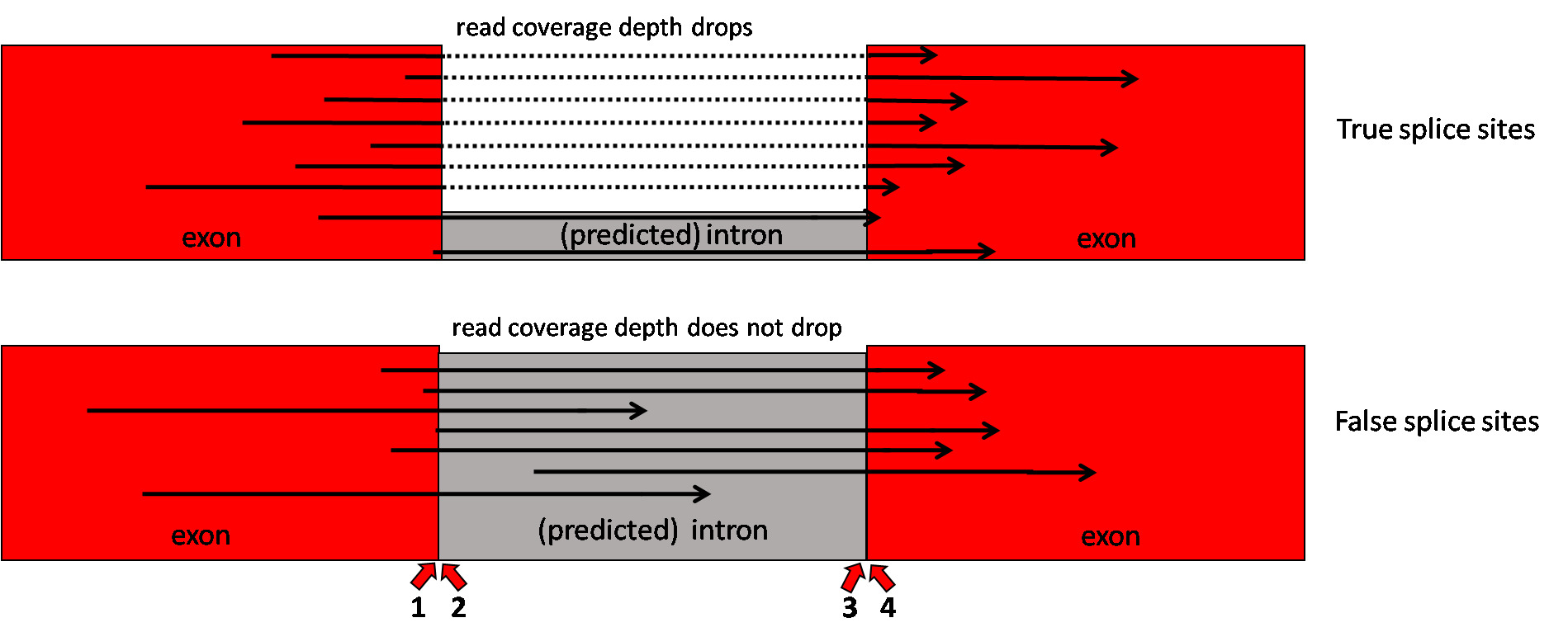

Supplement: Supplementary file 3 — RNA-Seq based splice site validation. Schematic illustration how the splitted mapping of RNA-Seq reads (arrows) over exons (red) and introns (grey) was used to validate splice sites. The read coverage depth should drop when moving from an exon into an intron. Red arrows indicate the four positions considered for this analysis. (JPG 168 kb) [file 12864_2018_5360_MOESM3_ESM.jpg]

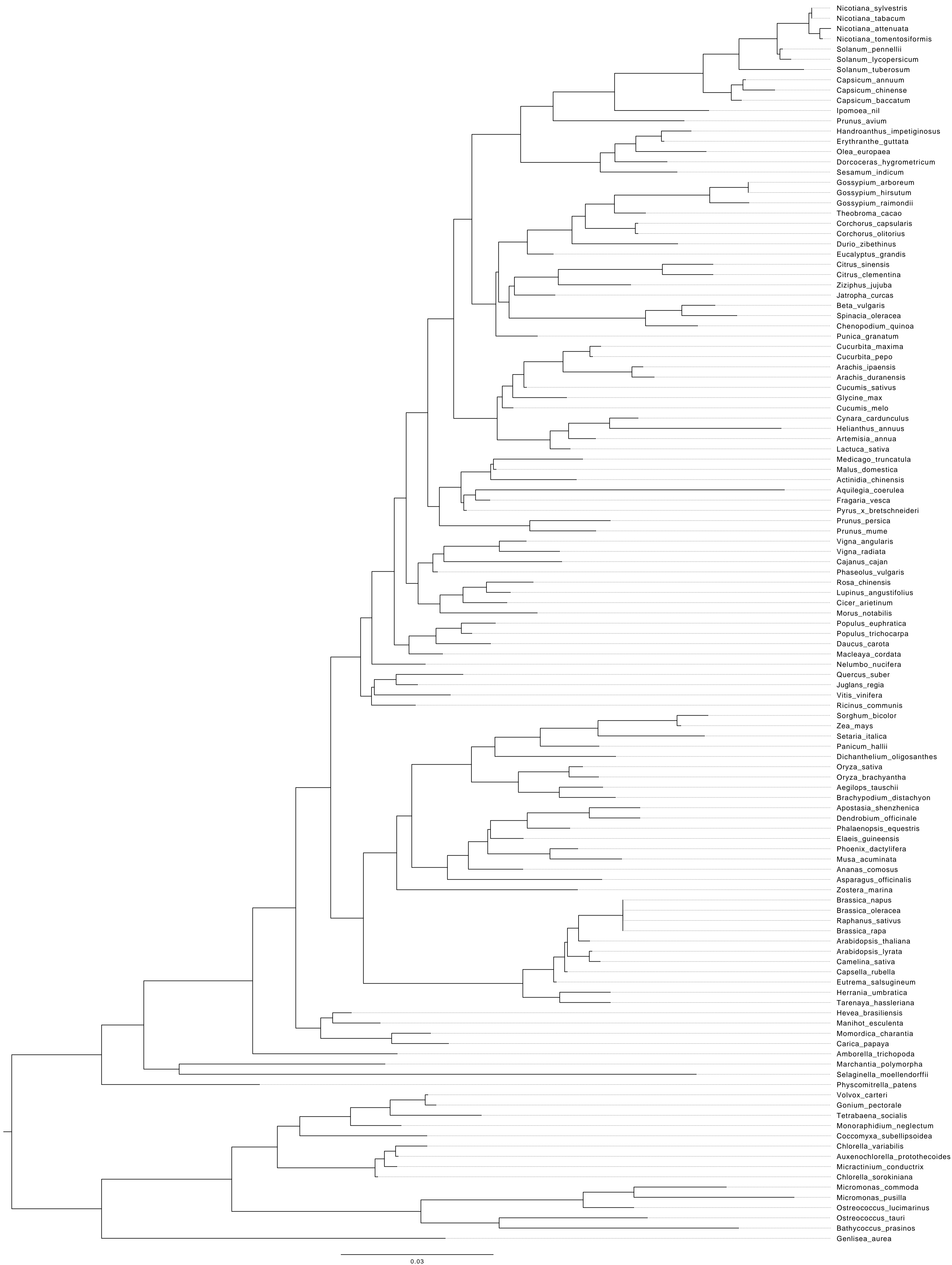

Supplement: Supplementary file 4 — Phylogenetic tree. RbcL sequences were used to construct a phylogenetic tree of all species involved in the analysis. Missing data points were corrected by relying on the NCBI taxonomy thus the branch lengths are not to scale. (PDF 8 kb) [file 12864_2018_5360_MOESM4_ESM.pdf]

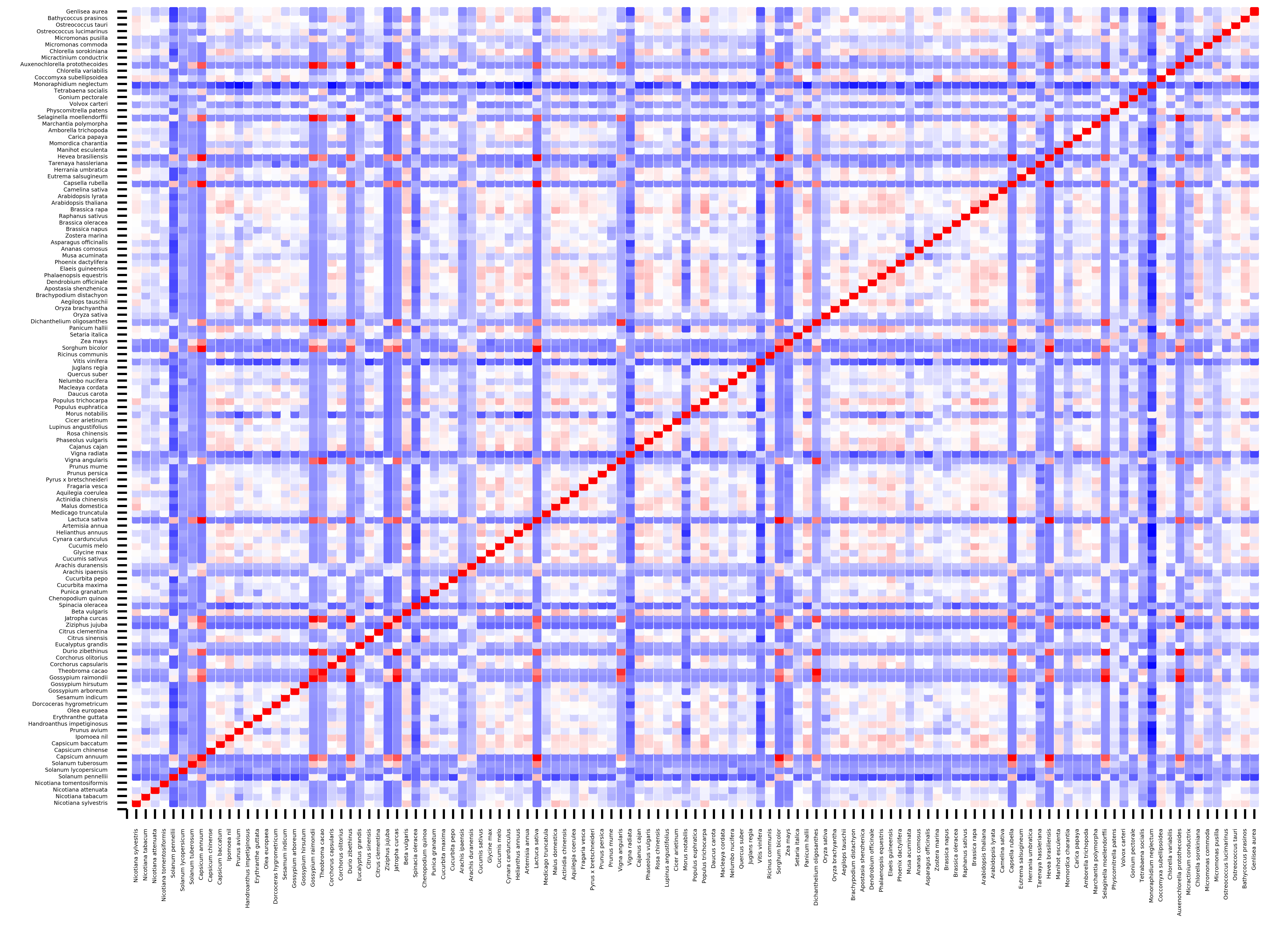

Supplement: Supplementary file 8 — Similarity of the non-canonical splice site pattern across plants. The Spearman correlation coefficient between each pair of species was calculated based on the observed frequency of all possible splice site combinations. Red color indicates similarity while blue color indicates substantial differences. As this correlation calculation takes the individual counts for all splice sites combinations in all species into account, it is possible to calculate correlation values even in the absence of non-canonical splice sites. (JPG 4157 kb) [file 12864_2018_5360_MOESM8_ESM.jpg]

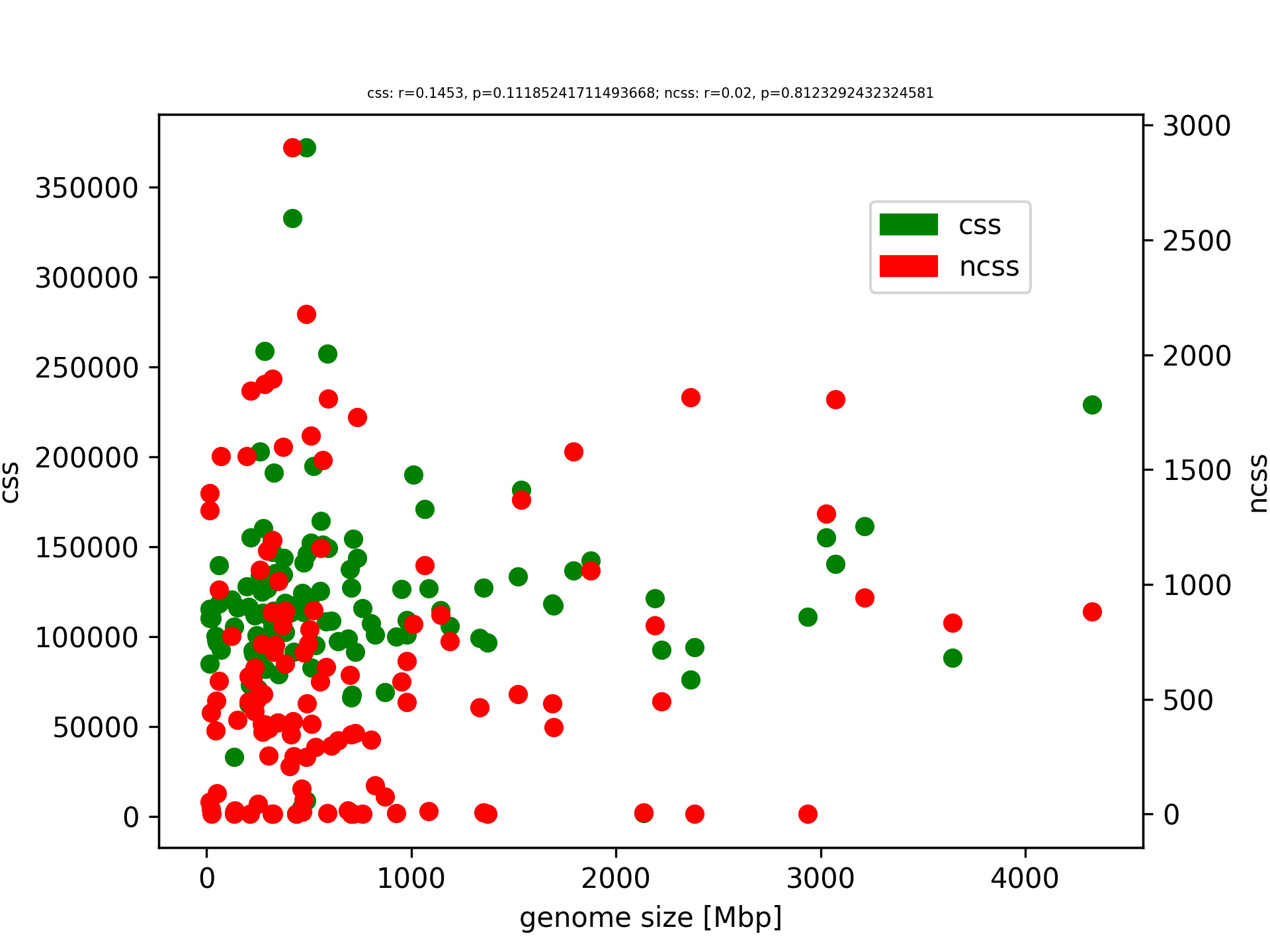

Supplement: Supplementary file 9 — Correlation of splice site frequencies with genome size. For each investigated species the number of canonical and non-canonical splice sites is displayed. The Spearman correlation coefficient between splice site number and genome size is r = 0.14 for canonical splice sites and r = 0.02 for non-canonical splice sites. (JPG 250 kb) [file 12864_2018_5360_MOESM9_ESM.jpg]

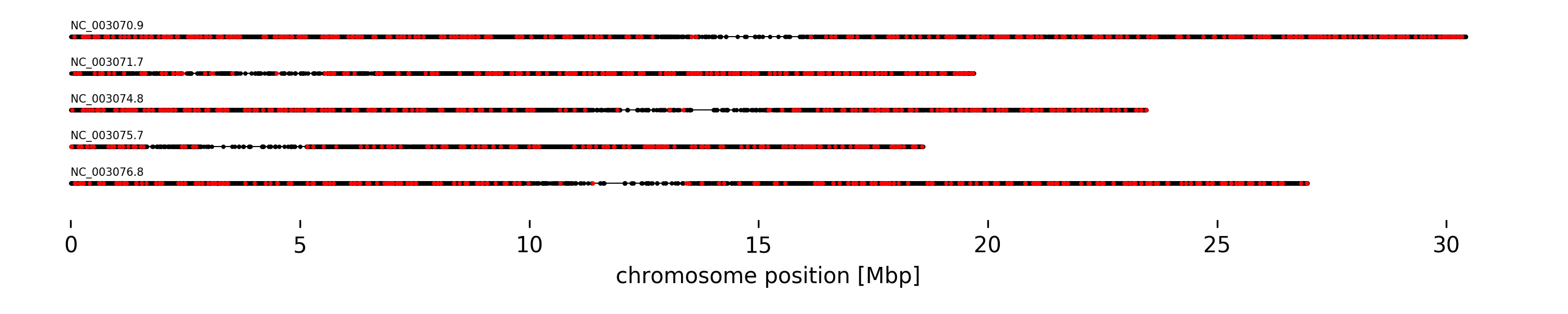

Supplement: Supplementary file 10 — Genome-wide distribution of non-canonical splice sites in A. thaliana. The distribution of genes with non-canonical splice sites (red dots) across the five chromosome sequences (black lines) of A. thaliana was analysed. (JPG 211 kb) [file 12864_2018_5360_MOESM10_ESM.jpg]

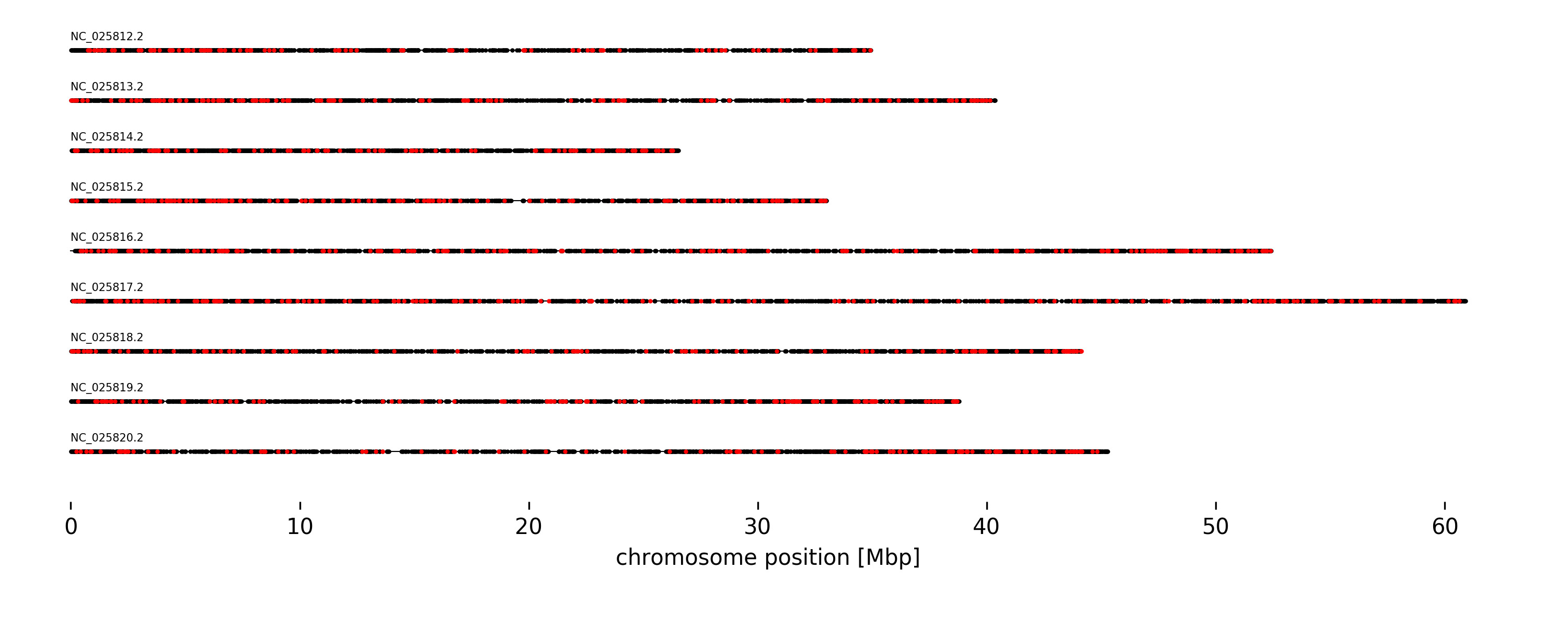

Supplement: Supplementary file 11 — Genome-wide distribution of non-canonical splice sites in B. vulgaris. The distribution of genes with non-canonical splice sites (red dots) across the nine chromosome sequences (black lines) of B. vulgaris was analysed. (JPG 339 kb) [file 12864_2018_5360_MOESM11_ESM.jpg]

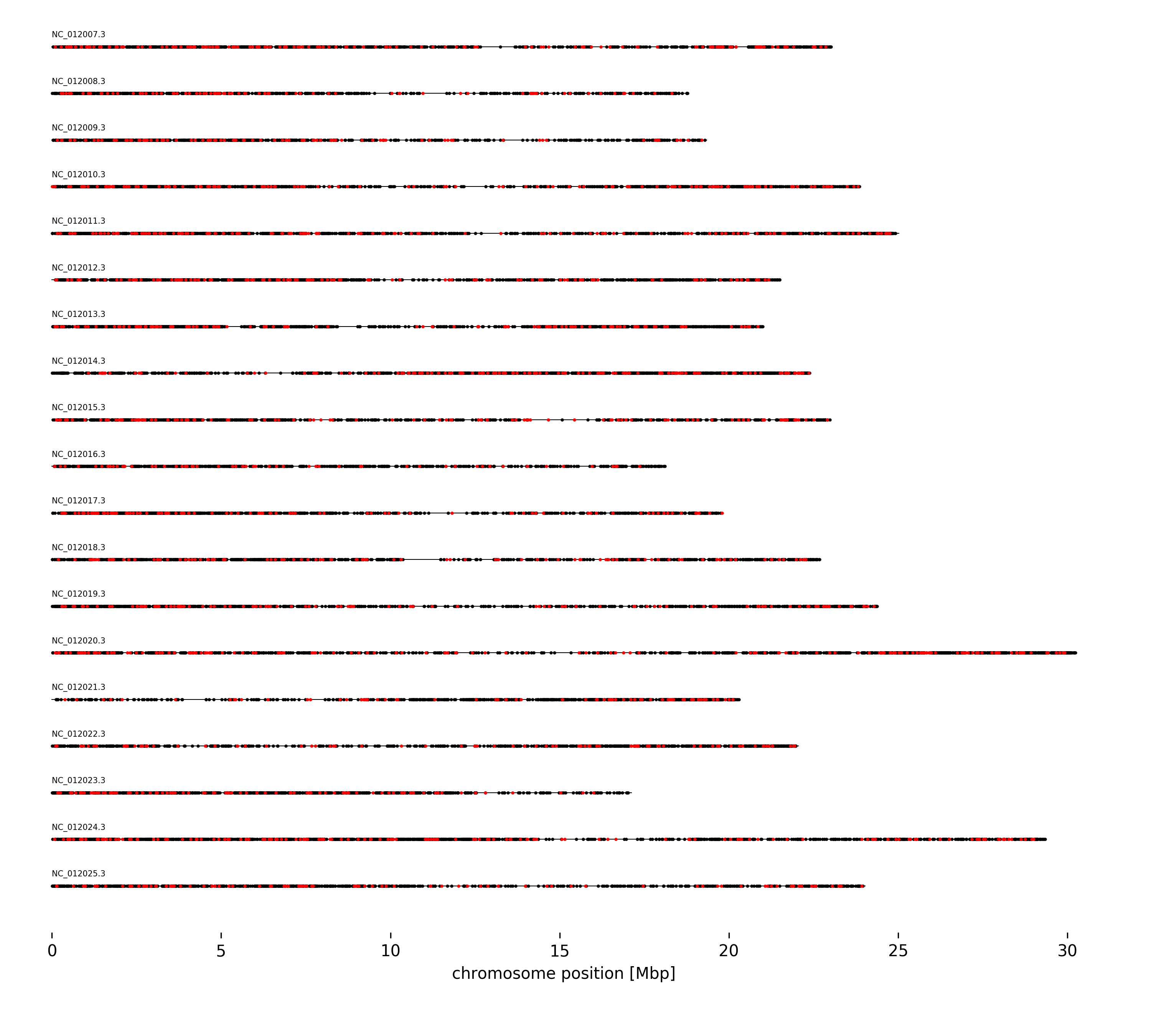

Supplement: Supplementary file 12 — Genome-wide distribution of non-canonical splice sites in V. vinifera. The distribution of genes with non-canonical splice sites (red dots) across the 19 chromosome sequences (black lines) of V. vinifera was analysed. (JPG 700 kb) [file 12864_2018_5360_MOESM12_ESM.jpg]

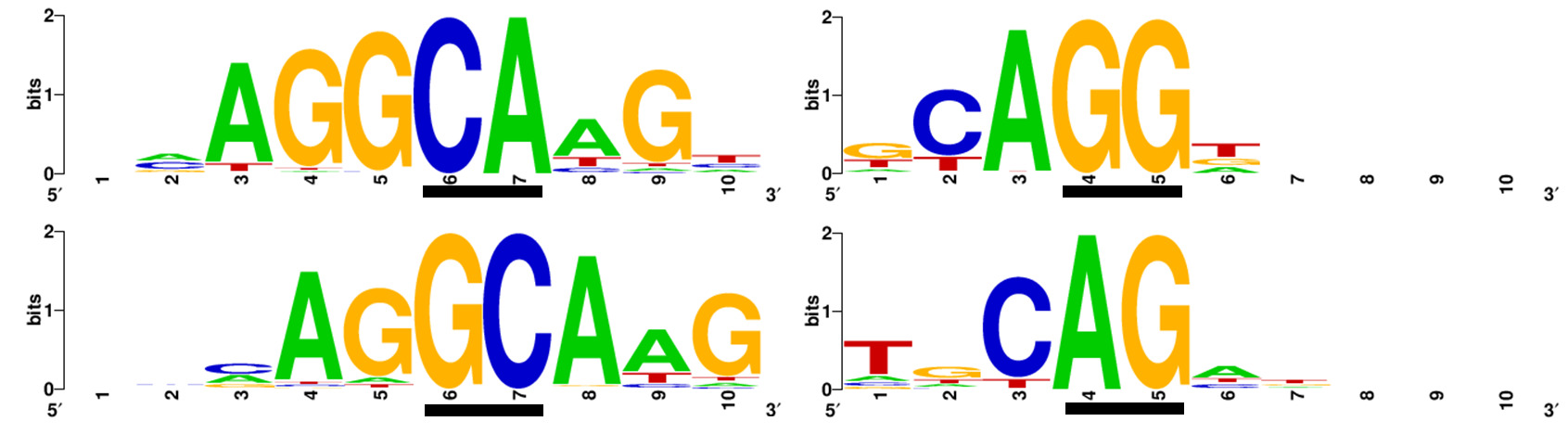

Supplement: Supplementary file 13 — Conserved sequences around splice sites in Oryza sativa. Predicted splice site combinations observed in Oryza sativa are indicated by a black line below them. Donor splice sites are on the left, acceptor splice sites on the right. The minor non-canonical splice combination CA-GG at the top could be converted into the major non-canonical GC-AG combination by just shifting one nucleotide to the left. The presence of two Gs at the acceptor splice site seems to correlate with the prediction of this CA-GG splice site combination instead of a major non-canonical GC-AG. (JPG 130 kb) [file 12864_2018_5360_MOESM13_ESM.jpg]

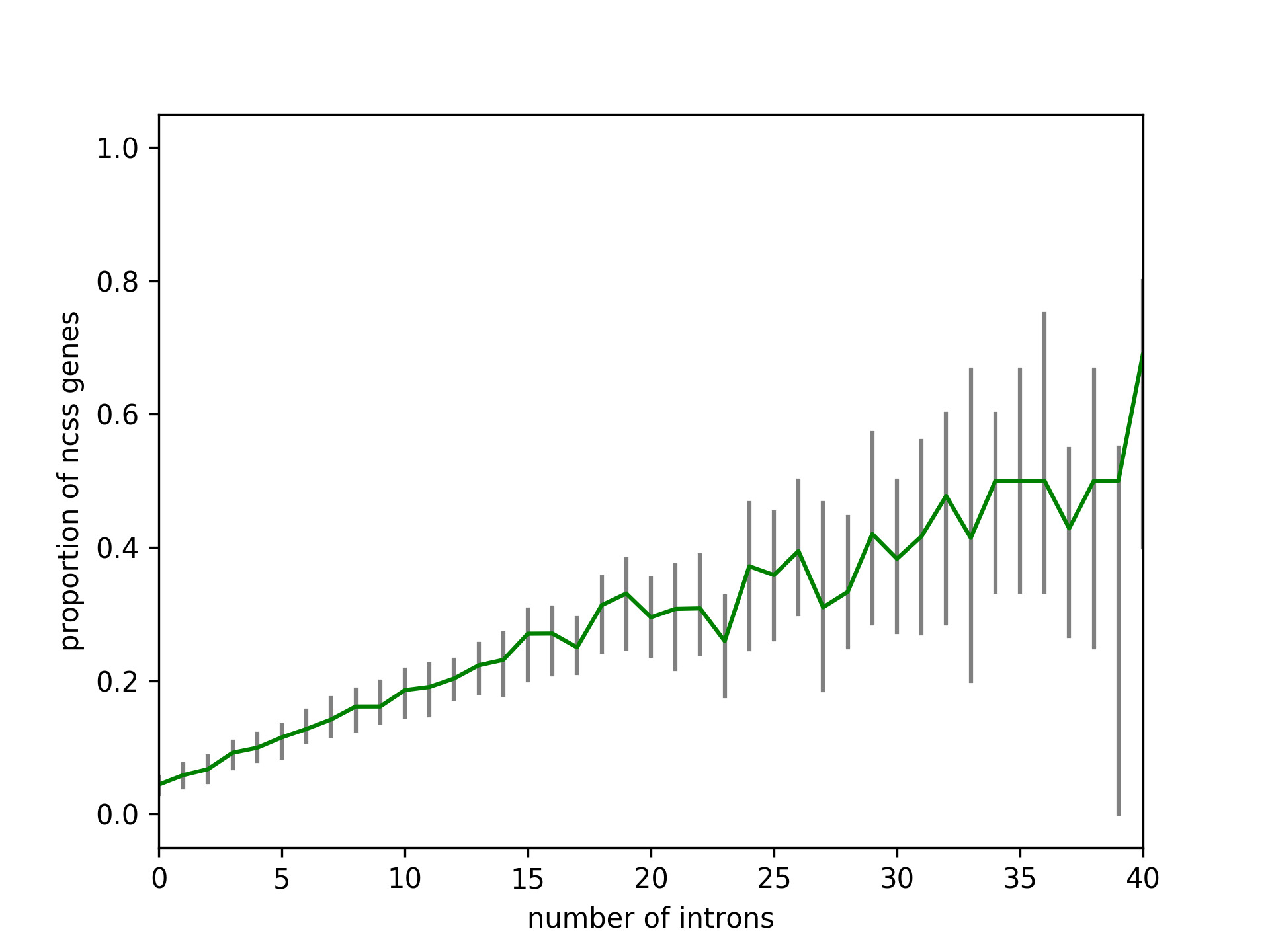

Supplement: Supplementary file 15 — Proportion of non-canonical splice sites. The green line indicates the average (median) proportion of genes with a non-canonical splice site combination. Grey lines indicate the range between 25 and 75% quantiles. Genes with more introns are more likely to have a non-canonical splice site combination. There is an almost perfect correlation up to 40 introns per gene. Insufficient sample sizes above this intron number prevent further analyses. (JPG 133 kb) [file 12864_2018_5360_MOESM15_ESM.jpg]

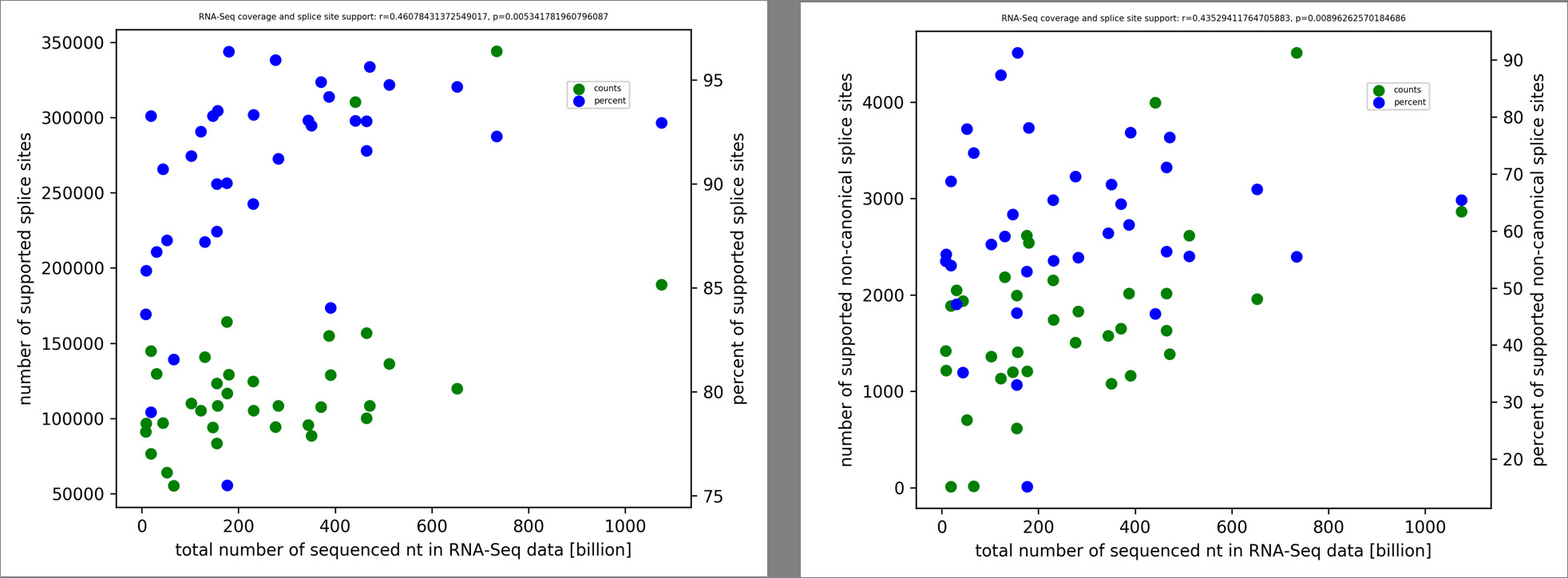

Supplement: Supplementary file 18 — RNA-Seq data set sizes. There is a moderate correlation between the amount of bases in the used RNA-Seq data sets and the number of supported splice sites. The trend is similar for canonical (r = 0.46) and non-canonical (r = 0.43) splice site combinations. (JPG 184 kb) [file 12864_2018_5360_MOESM18_ESM.jpg]
